# Rescue percutaneous coronary intervention for sinus node dysfunction following left atrial flutter ablation

**DOI:** 10.1016/j.hrcr.2021.04.015

**Published:** 2021-05-04

**Authors:** Yuichiro Miyazaki, Nobuhiko Ueda, Fumiyuki Otsuka, Koji Miyamoto, Teruo Noguchi, Kengo Kusano

**Affiliations:** ∗ Department of Cardiovascular Medicine, National Cerebral and Cardiovascular Center, Osaka, Japan; † Department of Advanced Cardiovascular Medicine, Graduate School of Medical Sciences, Kumamoto University, Kumamoto, Japan

**Keywords:** Anterior line, Percutaneous coronary intervention, Radiofrequency ablation, Sinus node dysfunction, Sinus nodal artery

## Introduction

Perimitral flutter (PMFL) is a common arrhythmia after ablation of atrial fibrillation. In some cases of PMFL, an anterior line is selected instead of the mitral isthmus line owing to technical and anatomical issues.^1^^,^^2^ In such cases, the sinus nodal artery (SNA) courses high on the anterior left atrium (LA) and is close to the block line on the anterior wall of the LA.^3^ Hence, the SNA could be injured by radiofrequency (RF) ablation leading to sinus node dysfunction (SND).^4^^,^^5^ Percutaneous coronary intervention (PCI) for SND due to SNA occlusion after stent implantation has been reported.^6^ However, few reports have shown the efficacy of the interventional strategy for SNA injury after RF application.

We report a case of SND following RF application on the anterior wall of the LA that was successfully managed using PCI for the occluded SNA.

## Case report

**Figure 1:**
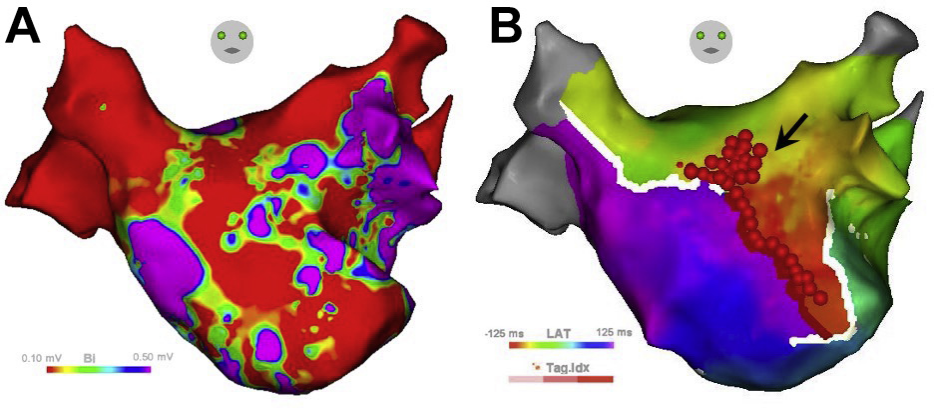
The voltage and activation map of the tachycardia and ablation points. **A:** The voltage map of the left atrium (LA) showed a broad low-voltage area at the anterior wall of the LA during sinus rhythm. **B:** The activation map showed tachycardia as a perimitral flutter. An anterior block line was performed between the mitral annulus and the right pulmonary vein (*red tag*). Additional radiofrequency application (*black arrow*) was delivered near the ostium of the LA appendage.

**Figure 2:**
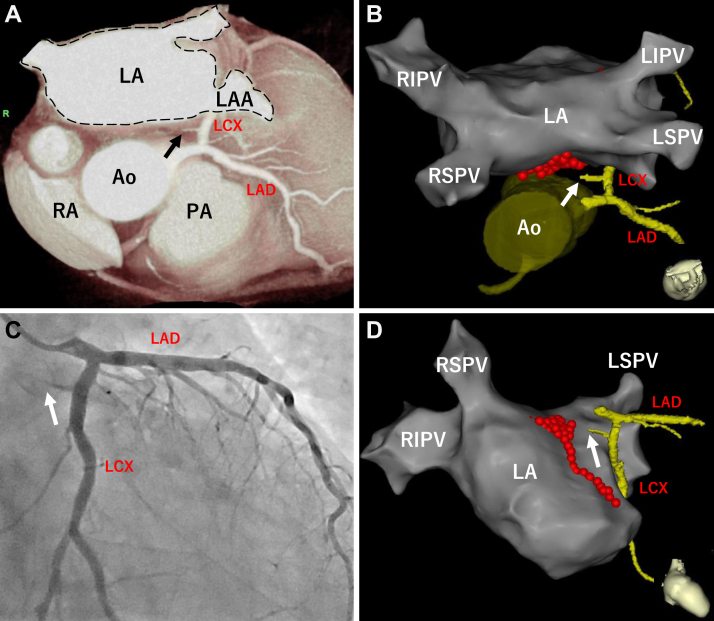
The contrast between coronary computed tomographic angiography (CCTA) or coronary angiography and the 3-dimensional map of the left atrium (LA). **A:** Volume-rendered view of CCTA showing occlusion of the sinus nodal artery (*black arrow*) originating from the left circumflex artery (LCX). **B:** The fusion of CCTA showing the left coronary artery and the anatomical shell of the LA is presented. The sinus nodal artery (*white arrow*) was occluded near the ablation point of the left atrial anterior wall (*red tag*). **C:** Coronary angiography showed an occluded sinus nodal artery (*white arrow*) originating from the LCX. **D:** The fusion of CCTA showing the left coronary artery and the anatomical shell of the LA is presented. The sinus nodal artery (*white arrow*) was occluded near the ablation point of the left atrial anterior wall (*red tag*). Ao = aorta; LAA = left atrial appendage; LAD = left anterior descending artery; LIPV = left inferior pulmonary vein; LSPV = left superior pulmonary vein; PA = pulmonary artery; RA = right atrium; RIPV = right inferior pulmonary vein; RSPV = right superior pulmonary vein.

A 66-year-old male patient with a history of 3 catheter ablations for paroxysmal atrial fibrillation was referred to our hospital for recurring atrial tachyarrhythmia; clinical arrhythmia was induced by an overdrive in atrial pacing. During repeat ablation, 3-D electroanatomical mapping revealed that the earliest atrial activation site in sinus rhythm was the junction of the superior vena cava and right atrium. Using a multielectrode catheter (PentaRay; Biosense-Webster, Inc, Diamond Bar, CA), 3-D mapping of the LA revealed that the tachycardia was PMFL. As there was a broad low-voltage area in the anterior wall of the LA during sinus rhythm (Figure 1), linear ablation from the mitral annulus to the right superior pulmonary vein was selected instead of the mitral isthmus line. Using an irrigated tip catheter (ThermoCool SmartTouch SF; Biosense-Webster, Inc), catheter ablation was performed on the anterior wall of the LA between the mitral annulus and right superior pulmonary vein (power, 35 W). However, the PMFL could not be terminated. Mapping of the LA using a multielectrode catheter revealed a gap in the PMFL circuit near the ostium of the LA appendage. As the circuit coursed into intramural LA, additional RF application (power, 40 W) was delivered near the ostium of the LA appendage, and the PMFL terminated to sinus rhythm (Figure 1). A few minutes after the final RF application, sinus rhythm disappeared and junctional rhythm appeared without significant ST changes. Although the sedative agent was stopped and continuous dopamine infusion (5.0 μg/kg per minute) was started for bradycardia and hypotension, sinus rhythm did not return. In view of irregular sinus node function (SNF) even after 2 days, coronary computed tomographic angiography was performed; it revealed occlusion of the SNA (Figure 2).

**Figure 3:**
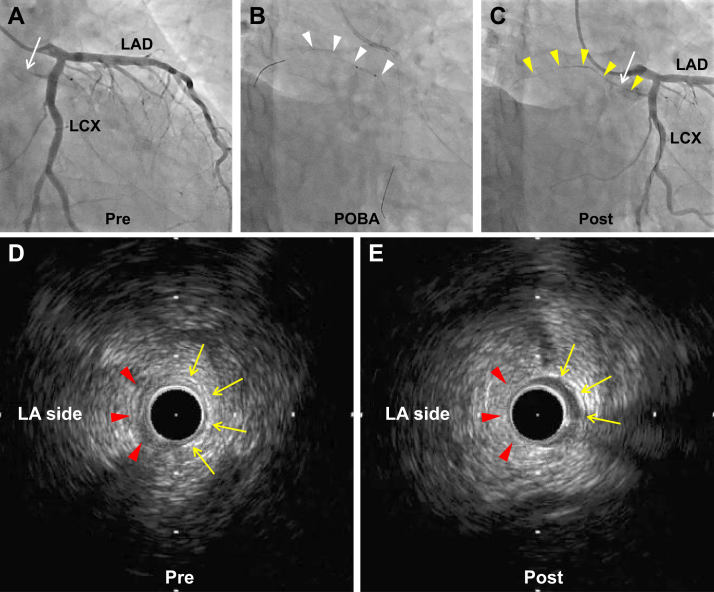
Percutaneous coronary intervention (PCI) for the occluded sinus nodal artery. **A:** Pre-PCI coronary angiography (CAG) showing the occluded sinus nodal artery (*white arrow*) originating from the left circumflex coronary artery (LCX). **B:** Plain old balloon angioplasty (POBA) using semi-compliant balloon (diameter = 1.5mm) for the occluded sinus nodal artery. *White arrowheads* indicate balloon-treated sites. **C:** Post-PCI CAG showing the recanalized sinus nodal artery (*yellow arrowheads*). **D:** Pre-PCI intravascular ultrasound (IVUS) cross-section showing the occluded lesion of the sinus nodal artery (corresponding with *white arrow* in panel A). The vessel diameter was approximately 1.5 mm and the lumen was invisible. The perimedial high-echoic band was partially identified (*yellow arrows*), while the vessel structure (3 layers of the vessel) on the left atrium (LA) side could not be clearly identified (*red arrowheads*). The orientation of the LA side was determined based on the location of the small side branches of the sinus nodal artery also identified on angiography. **E:** Post-PCI IVUS cross-section showing the recanalized lumen of the sinus nodal artery (corresponding with *white arrow* in panel C). The vessel structure was partially identified (*yellow arrows*), although the structure on the LA side remained to be determined (*red arrowheads*). The echogenicity of the adventitia on the LA side was relatively low. LAD = left anterior descending artery.

Coronary angiography was performed; it showed that the occluded SNA predominantly originated from the left circumflex artery and remained occluded even after intracoronary nitroglycerin administration (Figure 2). Subsequently, PCI was performed, and a hydrophilic polymer-jacketed 0.014- to 0.010-inch tapered guide wire (XT-R; ASAHI INTECC CO, LTD, Aichi, Japan) was successfully advanced distal to the occluded site of the SNA. Intravascular ultrasound (IVUS) revealed the absence of a 3-layered structure in the vessel wall on the LA side at the occluded site, where the echogenicity of the adventitia was relatively low. After balloon angioplasty using a 1.5 × 10 mm semi-compliant balloon (ZINRAI; Kaneka Corporation, Tokyo, Japan), the SNA was successfully recanalized (Figure 3). SNF recovered immediately after PCI, allowing termination of the dopamine infusion. No bradycardia, sinus pause, or atrial tachyarrhythmia occurred after PCI.

## Discussion

Acute SND can occur following linear ablation on the anterior wall of the LA, as shown in previous reports; the mechanism was thought to be direct thermal injury to the SNA.^4^^,^^5^^,^^7^ However, the precise mechanism underlying the thermal injury remains unclear; IVUS images obtained in this case indicate that RF application may lead to the partial destruction of the SNA vessel wall, resulting in arterial occlusion. The presence or absence of a luminal thrombus could not be determined using IVUS owing to the small size of the SNA, while a relatively low echogenicity of the adventitia on the LA side potentially indicated degeneration of the adventitial tissue after ablation, with or without interstitial edema.

SNA injury is more likely to result in SND if the SNA is the sole supply to the sinus node. The prevalence of an exclusively left-sided SNA is 30%–40%, and more than half run high on the anterior LA; this could be a risk factor for SND in cases of anterior LA ablation.^3^ It is important to determine the anatomy of the SNA and limit energy delivery to avoid SND after RF ablation.^5^^,^^8^ Nevertheless, creating an anterior line cannot be achieved without high RF energy in cases with PMFL circuits that involve fibers of the Bachmann bundle, an epicardial structure.^8^

The sinus node is reported to be resistant to anoxia as well as to increases in extracellular potassium^9^^,^^10^; however, the detailed mechanism is not well understood. As a previous study reported that the atrial pacing rate ranged from less than 1% to 99% (approximately half of the cases did not depend on atrial pacing) after pacemaker implantation for SND after ablation for paroxysmal atrial fibrillation, SNF appears to be reversible.^5^ The collateral vessels supplying blood to the sinus nodal cells could be one of the causes.^11^ Since SNF is reversible, SND after catheter ablation may resolve spontaneously within a few days.^5^^,^^8^ However, previous reports have shown that SND frequently fails to recover completely after catheter ablation and requires permanent pacing.^5^ That may be because the SNA of patients in those reports was not recanalized. The current case, for the first time, demonstrates the successful recovery of a postablation SND using PCI for the occluded SNA. Recanalization of the SNA was effective 2 days after catheter ablation. Our case shows that PCI can help recovery from SND and avoid permanent pacing, especially in cases requiring high RF for ablation on the anterior wall of the LA.

## Conclusion

The current case showed that SND during the ablation procedure, especially at the anterior wall of the LA, can cause SNA injury; PCI for the occluded SNA can be a useful strategy to recover SNF and avoid permanent pacing.

**Key Teaching Points**

• The sinus nodal artery (SNA) coursing along the high anterior left atrium (LA) can be injured by radiofrequency (RF) application to the LA anterior wall, leading to sinus node dysfunction.
• The intravascular ultrasound images obtained in the present case indicate that RF application may lead to the partial destruction of the vessel wall structure of the SNA, resulting in arterial occlusion.
• Percutaneous coronary intervention for occluded SNA could be a useful strategy to recover sinus node function and avoid permanent pacing.
